# The Use of Nanorobotics in the Treatment Therapy of Cancer and Its Future Aspects: A Review

**DOI:** 10.7759/cureus.29366

**Published:** 2022-09-20

**Authors:** Muskan Aggarwal, Sunil Kumar

**Affiliations:** 1 Medicine, Jawaharlal Nehru Medical College, Datta Meghe Institute of Medical Sciences, Wardha, IND

**Keywords:** nanorobots, nanorobotics, nanogenerators, drug delivery system, chemotherapy, cancer therapy, nanotechnology

## Abstract

The late Nobel Physicist Richard P. Feynman, in a dinner talk in 1959, very rightly said that there is enough room for the betterment of technology beyond our scope of imagination, proposing utilizing mechanical tools to make those that are relatively smaller than the others, which further can be rendered fruitful in making even more compact mechanical devices, all the way down to the level of the smallest known atom, emphasizing that this is "a progress which I believe cannot be avoided". Feynman proposed that nanomachines, nanorobots, and nanodevices may eventually be utilized to construct a huge range of atomically accurate microscopic instruments and manufacturing equipment, as well as a large number of ultra-small devices and other nanoscale and microscale robotic structures. Biotechnology, molecular biology, and molecular medicine could be used to create totally self-sufficient nanorobots/nanobots. Nanorobotics includes sophisticated submicron devices constructed of nanocomponents that are viewed as a magnificent desired future of health care. It has a promising potential in medication delivery technology for cancer, the top cause of mortality among those under the age of 85 years. Nanorobots might transport and distribute vast volumes of anticancer medications into diseased cells without hurting normal cells, decreasing the adverse effects of existing therapies such as chemotherapy damage. The ultimate development of this innovation, which will be accomplished via a close partnership among specialists in robotics, medicine, and nanotechnology, will have a significant influence on illness detection, therapy, and prophylaxis. This report includes a study on several ways to cancer therapy utilizing nanorobots. Furthermore, it offers insight into the future breadth of this area of research.

## Introduction and background

Researchers have emphasised nanotechnology as an outstanding technological trend in the last few decades, and it is characterized by the fast proliferation of electronics for applications in communication, known as nanomedicine, and environmental monitoring. Studies are now being conducted on the scientific bottlenecks that affect the lifespan of the living, particularly humans. Among these constraints are illnesses with few or no alternatives for treatment and healing. A drug delivery system (DDS) refers to an alternative diagnosis and/or therapy that has been shown in the medical fraternity [1,2]. Nanorobots are nanoelectromechanical systems (NEMS), a recently developed chapter in miniaturisation, similar to microelectromechanical systems (MEMS), which is already a multibillion-dollar business. Designing, architecting, producing, programming, and implementing such biomedical nanotechnology are all part of nanorobotics and NEMS research. Any scale of robotics includes calculations, commands, actuation and propulsion, power, data-sharing, interface, programming, and coordination. There is heavy stress on actuation, which is a key prerequisite for robotics [1]. The similarity in size of nanorobots to that of organic human cells and organelles brings up a huge variety of its possible uses in the field of health care and environmental monitoring of microorganisms. Other potential uses, such as cell healing, may be possible if nanorobots are tiny enough to reach the cells. Furthermore, it is still to be realised that the tiny sensors and actuators' square measures are necessary for the growing concept of a strongly connected ascending information technology infrastructure; the envision of artificial cells (nanorobots) that patrol the cardiovascular system, thus, detecting and destroying infections in minute quantities. This might be a programmable system with approachable ramifications in medicine, creating a revolutionary replacement from therapy to bar [1]. Chemotherapeutic substances employed in cancer treatment measure disseminates non-specifically throughout the body, where they exert an influence on both malignant and normal cells, restricting the drug quantity feasible within the growth and also resulting in unsatisfactory medication due to excessive toxic hazards of the chemotherapy drugs on normal cells of the body. It is safe to say that molecularly focused medical care has evolved as a collaborative method to overcome the lack of specificity of traditional cancer therapy drugs [3]. With the help of nanotechnology, intercellular aggregation of the drugs in cancer cells can be increased while minimising the risk of unwanted drug toxicity in normal cells by utilising various drug targeting mechanisms [4].

## Review

This review article focuses on the recent advancements, technological growth, and expansion in the field of nanorobotics and nanotechnology and its application in the discipline of bio-healthcare systems, principally for the DDS in the medication of cancer. Existing research literature and relevant studies regarding the topic of concern were read and a detailed analysis was undertaken in the indexes of PubMed, Science Direct, MEDLINE, Scopus, and Google Scholar. Hardly any language or time constraints were applied. To obtain a detailed search, more articles, synonyms, and derivatives of the phrases were employed; the following evaluation phrases were used: "drug delivery", "cancer", "neoplasms", and "cancer therapy".

Nanorobots and their types

Nanorobots are miniaturised machines that have the ability to perform work at par with that of current existing machines, having applications in the aspects of medicine, industry, and other areas like the development of nanomotors employed for the conservation of energy; nanorobots have also proved to be serviceable in reducing infertility problems by acting as an engine and giving a boost to the sperm motility when attached to them [2]. Organic and inorganic nanorobots are by far the most commonly studied. Organic nanorobots, also known as bio-nanorobots, are created by combining virus and bacterium DNA cells. This type of nanorobot is less harmful to the organism. Diamond structures, synthetic proteins, and other materials are used to make inorganic nanobots, which are more hazardous than organic nanobots. To overcome this hurdle of toxicity, researchers have devised a way involving encapsulating the robot, thus decreasing its chances of being destructed by the body's self-defence mechanism [5,6]. Scientists can gain an understanding of how to energise micro and nano-sized devices using reactionary processes if they understand the biological motors of live cells [7]. The Chemistry Institute of the Federal Fluminense University created a nano valve, which is made up of a tank covered with a shutter in which dye molecules are housed and may leave in a uniform fashion whenever the cover is opened. This gadget is also natural, made of silica (SiO2), beta-cyclodextrins, and organo-metallic molecules, and shall be used in therapeutic applications [1]. Proteins are employed in certain studies to feed nanomotors that can move huge objects, as well as the use of DNA hybridisation and antibody protein in the development of nanorobots. DNA hybridisation is defined as a process by which two complementary single-stranded DNA and/or RNA molecules bond together to form a double-stranded molecule. A nanorobot can be functionalized using a variety of chemical compounds [8]. It has been investigated in nanomedicine in DDS, which operates directly on targeted cells of the human body. Researchers create devices that can administer medications to precise places while simultaneously adjusting the dose and amount of release. This DDS using nanorobots can be used to treat joint disorders, dental problems, diabetes, cancer, hepatitis and other conditions [2,9-12]. One of the benefits of this technology is the potential to diagnose and treat illnesses with minimal impact on normal tissues, minimizing the likelihood of negative effects and guiding healing and remodelling therapy at the cellular and sub-cellular levels [13,14].

Chemotherapy drug delivery using nanorobots in cancer treatment

**Figure 1:**
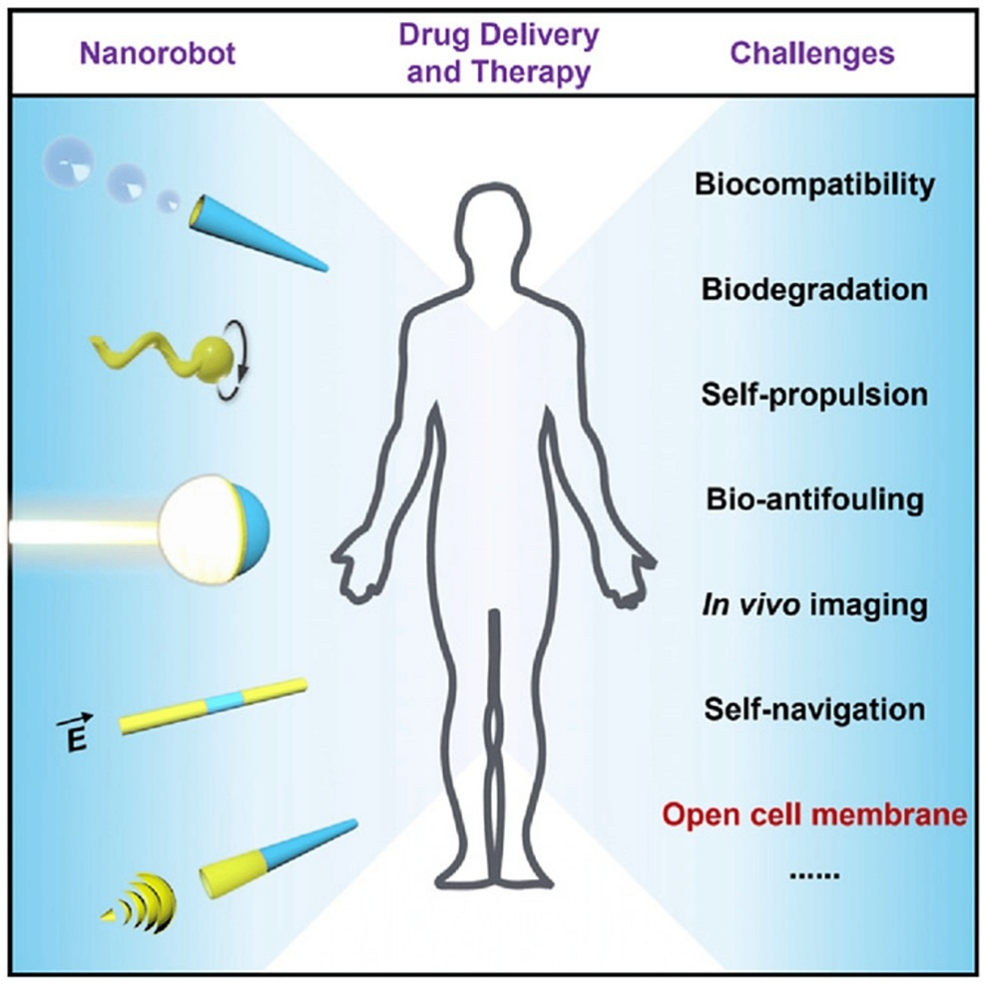
Challenges of nanorobots in drug delivery. This image throws light on the various challenges that different shaped nanorobots face when employed for drug delivery [38]. Image reprinted from [38] under Creative Commons Attribution 4.0 International License.

New advances in medication delivery have resulted in greater quality in targeted drug delivery that uses nanosensors to detect particular cells and regulate discharges through the use of smart medicines [1]. Traditional chemotherapeutic drugs act by eliminating swiftly replicating cells, which is a primary feature of malignant cells. Most anticancer medications have a limited therapeutic boundary, often resulting in cytotoxicity to normal stem cells that proliferate quickly, such as bone marrow, macrophages, gastrointestinal tract (GIT), and hair follicles, causing adverse effects like myelosuppression (lower synthesis of WBCs, producing immunosuppression), mucositis (inflammation of the GIT lining), alopecia (hair loss), organ malfunction, thrombocytopenia/anaemia, and haematological side effects, among other things. Doxorubicin is used to treat numerous forms of cancer, including Hodgkin's disease, when it is combined with other antineoplastic medicines to minimize its toxicity [15,16]. Paclitaxel is a drug that is injected intravenously and is used to treat breast cancer. Some of the significant side effects include bone marrow suppression and progressive neurotoxicity. Cisplatin is an alkylating drug that results in the intra-DNA binding filament. Its negative effects include giddiness and severe vomiting, and it can be nephrotoxic [1]. Camptothecin is applied to treat neoplasia by inhibiting type 1 topoisomerases, an enzyme required for cellular duplication of genetic information. Numerous initiatives have been launched with the goal of employing nanotechnology to build DDS that can reduce the negative impacts of traditional therapy. On the surface of single-walled carbon nanotubes (SWNTs), doxorubicin was layered [17]. Doxorubicin was used in metastatic tumour cells as a polymer prodrug/collagen hybrid. The use of polymeric pro-drug nanotechnology in the therapy of rapidly dividing abnormal cells is a novel advance in the field [18]. Nanotechnology is continually looking for biocompatible materials that may be used as a DDS. The nanoparticle hydroxyapatite (HA), a significant component of bone and teeth, was employed to deliver paclitaxel, an anti-neoplastic medication, and the out-turn implies that therapy should begin with hydrophobic medicines [19]. Various initiatives have been launched with the goal of employing nanotechnology to build DDS, which can reduce the negative influence of traditional chemotherapy. The limitation of conservative chemotherapeutics is that it is unable to target malignant cells exclusively. These above-listed adverse effects often result in a delay in treatment, reduced drug dose or intermittent stopping of the therapy [20]. Given the ability of nanorobots to travel as blood-borne devices, they can aid in crucial therapy procedures such as early diagnostics and smart medication administration [21]. A nanorobot can aid with smart chemotherapy for medication administration and give an efficient early dissolution of cancer by targeting only the neoplastic-specific cells and tissues and preventing the surrounding healthy cells from the toxicity of the chemotherapy drugs so being used. Nanorobots as drug transporter for timely dose administration allow chemical compounds to be kept in the bloodstream for as long as essential, giving expected pharmacokinetic characteristics for chemotherapy in the therapies for anti-cancer as shown in Figure 1 [22-25]. The clinical use of nanorobots for diagnostic, therapy, and surgery can be accomplished by injecting them via an intravenous route. The nanorobots may be getting intravenously injected into the body of the recipient. The chemotherapy pharmacokinetics comprises uptake, metabolism, and excretion, as well as a rest period to allow the body to re-establish itself ahead of the succeeding chemotherapy session. For tiny tumours, patients are often treated in two-week cycles [26]. As a primary time threshold for medical purposes, nanorobots can be used to assess and diagnose the tumour within a short span of time using proteomic-based sensors. The magnetic resonance contrast-agent uptake kinetics of a very small molecular weight can forecast the transport of protein medicines to solid tumours [27]. Testing and diagnostics are critical components of nanorobotics study. It provides speedy testing diagnosis at the initial visit, eliminating the need for a follow-up appointment following the lab result, and illness identification at an earlier stage. The demand for energy for propulsion is a restriction in the usage of nanorobots in vivo. Because small inertia and strong viscous forces are associated with less productivity and less convective motion, higher quantities of energy are required [28]. Drug retention in the tumour will decide the medication's effectiveness after nanorobots pass cellular membranes for targeted administration. Depending on its structure, medication transport pathways from plasma to tissue impact chemotherapy to achieve more effective tumour chemotherapy [27]. According to the latest research, nanotechnology, DNA production of molecular-scale devices with superior control over shape, and site-specific functionalisation assures interesting benefits in the advancement of nanomedicine. However, biological milieu uncertainty and innate immune activation continue to be barriers to in vivo deployment. Thus, the primary benefit of nanorobots for cancer medicine administration is that they reduce chemotherapeutic side effects. The nanorobot design integrates carbon nanotubes and DNA, which are current contenders for the latest types of nanoelectronics, as the optimum method [29]. As a compound bio-sensor with sole-chain antigen-binding proteins, a complementary metal oxide semiconductor (CMOS) is used for building circuits with characteristic sizes in tens of nanometres [30]. For medicament release, this approach employs stimulation elicited upon proteomics and bioelectronics signals. As a result, nanoactuators are engaged to adjust medication delivery whenever the nanorobot detects predetermined modifications in protein gradients [1,31]. Thermal and chemical signal changes are relevant circumstances directly connected to significant medical target identification. Nitric oxide synthase (NOS), E-cadherin, and B cell lymphoma-2 (Bcl-2) are some instances of fluctuating protein aggregation within the body near a medical target under diseased conditions. Furthermore, temperature changes are common in tissues with inflammation [32]. The framework integrates chemical and thermal characteristics as the most essential clinical and therapeutic recommendations for nanorobot template analysis. It also integrates chemical and thermal characteristics as the most essential diagnostic and therapeutic recommendations for nanorobot framework evaluation. The simulation in a three-dimensional real-time setting attempts to provide a viable model for nanorobot foraging within the body. One of the breakthroughs describes a hardware structure rooted in nano-bioelectronics for the use of nanorobots in neoplasia therapy [33,34]. The continuous venture in building medical micro-robots has led to the initial conceptual framework research of a full medical nanorobot until now issued in a peer-reviewed publication, "Respirocytes", detailed a theoretical unnatural mechanical red blood cell, or "Respiro-cytes", consisting of 18 billion perfectly ordered architectural atoms proficient in delivering 236 times extra oxygen to the tissues and cells of the body per unit volume than normal red blood cells [35]. Microbivores, or unnatural phagocytes, might monitor the circulation, searching for and eliminating pathogens such as bacteria, viruses, or fungi. These nanobots may use up to 200 pW continuously. This capability is employed to break down germs that have been entrapped. Microbivores have biological phagocytic defences that are either organic or antibiotic-assisted, and they can operate up to 1,000 times quicker. Even the most serious septicaemic diseases will be eliminated by microbivores within a short span of time. Because virulent microorganisms are entirely digested into harmless sugars and amino acids, which are the nanorobot’s sole discharge, the nanorobots reject the advanced possibility of sepsis or septic shock [36,37].

Future of nanotechnology in the area of medicine

**Figure 2:**
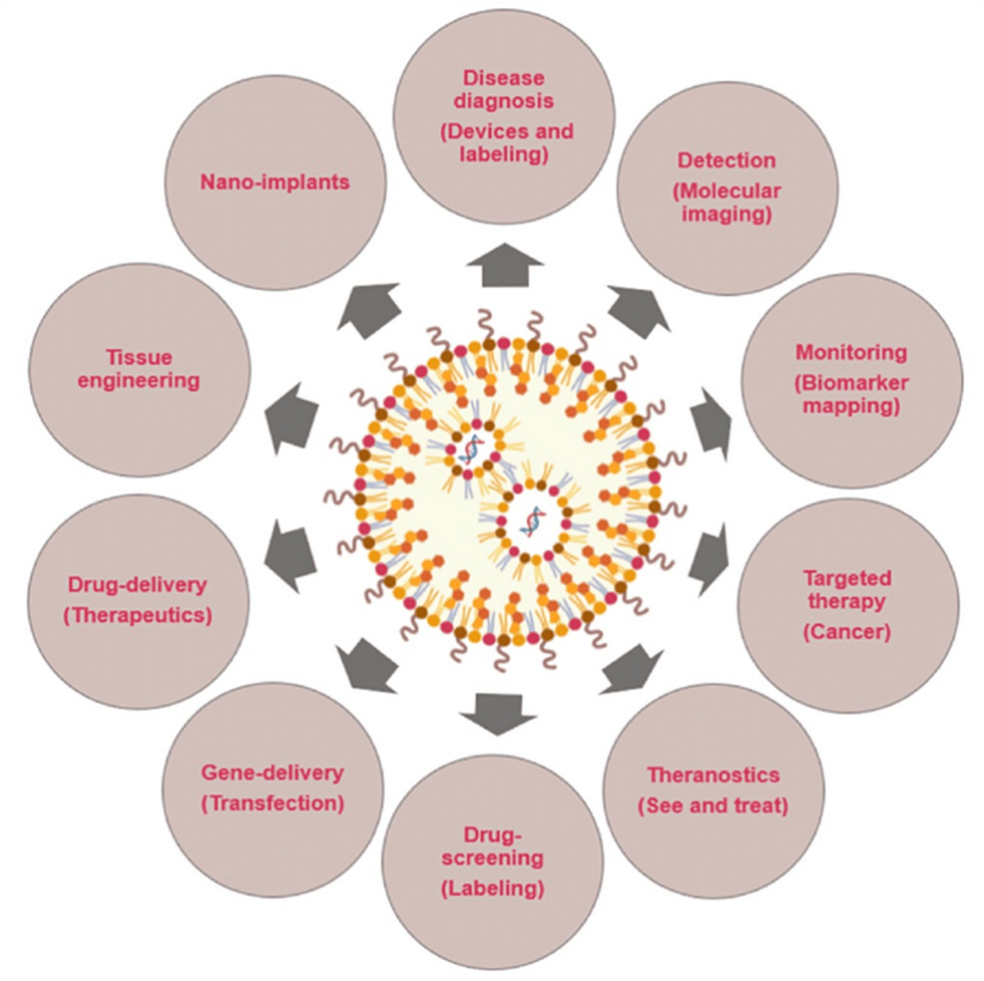
Illustration showing various other applications of nanotechnology in medicine. The discovery of nanoparticles with the help of nanotechnology has led to its various uses in the area of medicine. The nanoparticle so created can be employed for various uses like in the manufacturing of nano implants, tissue engineering for drug delivery systems, gene delivery systems, drug screening, theranostics, cancer therapy, biomarker mapping, disease detection, and bio-imaging [39]. Image reprinted from [39] under Creative Commons Attribution 4.0 International License.

**Figure 3:**
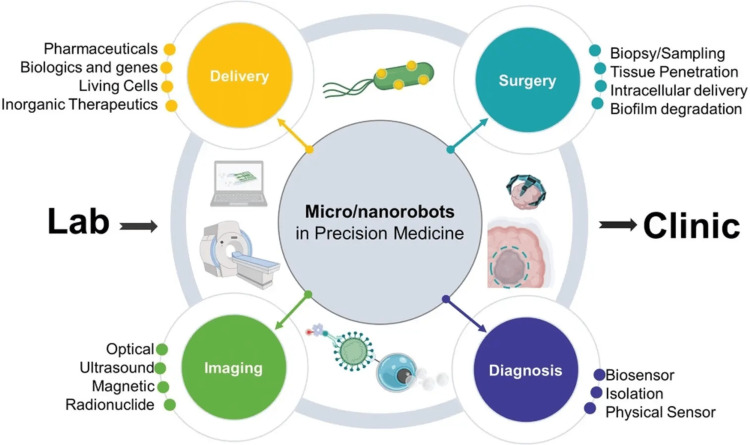
Schematic diagram of the current trends of micro/nanorobotics in precision medicine. Nanorobots are being used in various domains of pre-clinical and clinical medicine. In pre-clinical medicine, nanorobots are being employed in bioimaging and various delivery systems of drugs, gene therapy, living cells, and inorganic therapeutics. Similarly, nanorobots in clinical medicine are being extensively used in disease diagnosis and surgeries for biopsy, biofilm degradation, tissue collection, and sampling [41]. Image reprinted from [41] under Creative Commons Attribution 4.0 International License.

To bring in combination the required collaborative skills to produce these unique technologies, numerous conventional streams of science, such as medicine, chemistry, physics, materials science, and biology, have come together to form the expanding field of nanotechnology. Nanotechnology has a vast span of possible applications (Figure 2) [39], from improvements to current practices to the creation of entirely new tools and skills. The last few years have observed an exponential increase of interest in the topic of nanotechnology and research, which has led to the identification of novel applications for nanotechnology in medicine and the emergence of an advanced branch called nanomedicine. It includes the science and technology of diagnosing, treating, and preventing illness, traumatic injury, and alleviating pain; conserving and enhancing human health using nanoscale architectured materials, biotechnology, and genetic engineering; eventually, complex machine systems and nanorobots, known as "nanomedicine" (Figure 3) [40,41].

In vivo diagnostics, nanomedicine might create technologies that can act within the human body to diagnose ailments earlier and identify and measure toxic chemicals and tumour cells. In the surgical aspect, when launched into the body through the intravenous route or cavities, a surgical nanorobot controlled or led by a human surgeon might work as a semi-autonomous on-site surgeon. An inbuilt computer might manage the device's operations, such as looking for disease and identifying and fixing injury by nanomanipulation while maintaining communication with the supervising surgeon via coded ultrasonic signals [37]. By transforming mechanical energy from bodily movement, muscle stretching, or water flow into electricity, scientists were able to design a new generation of self-sustained implanted medical devices, sensors, and portable gadgets [39]. Nanogenerators generate electricity by bending and then releasing piezoelectric and semiconducting zinc oxide nanowires. Nanowires may be produced on polymer-based films, and the utilization of flexible polymer substrates may one day allow portable gadgets to be powered by their users' movement [39]. Fluorescent biological labelling, medication and gene delivery, pathogen identification, protein sensing, DNA structure probing, tissue engineering, tumour identification, separation and purification of biological molecules and cells, MRI contrast enhancement, and phagokinetic research are among the uses. The extended duration effect of nanomedicine study is to describe quantitative molecular-scale components called nanomachinery. Accurate command and manipulation of nanomachinery in cells can lead to a more diverse and advanced gain in the interpretation of cellular processes in organic cells, as well as the creation of new technologies for disease detection and medication. The advantage of this research is the formation of a platform technology that will affect nanoscale imaging methodologies aimed to investigate molecular pathways in organic cells [40,42].

## Conclusions

The main target of writing this review was to provide an outline of the technological development of nanotechnology in medicine by making a nanorobot and introducing it in the medication of cancer as a new mode of drug delivery. Cancer is described as a collection of diseases characterised by the unregulated development and spread of malignant cells in the body, and the number of people diagnosed every year keeps adding up. Cancer treatment is most likely the driving force behind the creation of nanorobotics; it can be auspiciously treated using existing medical technology and therapeutic instruments, with the major help of nanorobotics. To decide the prognosis and chances of survival in a cancer patient, consider the following factors: better prognosis can be achieved if the evolution of the disease is time-dependent and a timely diagnosis is made. Another important aspect is to reduce the side effects of chemotherapy on the patients by forming efficient targeted drug delivery systems. Programmable nanorobotic devices working at the cellular and molecular level would help doctors to carry out precise treatment. In addition to resolving gross cellular insults caused by non-reversible mechanisms or to the biological tissues stored cryogenically, mechanically reversing the process of atherosclerosis, enhancing the immune system, replacing or re-writing the DNA sequences in cells at will, improving total respiratory capacity, and achieving near-instant homeostasis, medically these nanorobots have been put forward for use in various branches of dentistry, research in pharmaceuticals, and aid and abet clinical diagnosis. When nanomechanics becomes obtainable, the ideal goal of physicians, medical personnel, and every healer throughout known records would be realized. Microscale robots with programmable and controllable nanoscale components produced with nanometre accuracy would enable medical physicians to perform at the cellular and molecular levels to heal and carry out rehabilitating surgeries. Nanomedical doctors of the 21st century will continue to make effective use of the body's inherent therapeutic capacities and homeostatic systems, since, all else being equal, treatments that intervene the least are the best.
